# Spatial accessibility of general inpatient care in Germany: an analysis of surgery, internal medicine and neurology

**DOI:** 10.1038/s41598-020-76212-0

**Published:** 2020-11-05

**Authors:** Jan Bauer, Doris Klingelhöfer, Werner Maier, Lars Schwettmann, David A. Groneberg

**Affiliations:** 1 Division of Health Services Research, Institute of Occupational Medicine, Social Medicine and Environmental Medicine, Goethe University Frankfurt, Theodor Stern Kai 7, 60590 Frankfurt/Main, Germany; 2 Institute of Health Economics and Health Care Management, Helmholtz Zentrum München, 85764 Neuherberg, Germany; 3 Department of Economics, Martin Luther University Halle-Wittenberg, 06099 Halle an der Saale, Germany

**Keywords:** Health policy, Health services, Public health, Computer science

## Abstract

Improving spatial accessibility to hospitals is a major task for health care systems which can be facilitated using recent methodological improvements of spatial accessibility measures. We used the integrated floating catchment area (iFCA) method to analyze spatial accessibility of general inpatient care (internal medicine, surgery and neurology) on national level in Germany determining an accessibility index (AI) by integrating distances, hospital beds and morbidity data. The analysis of 358 million distances between hospitals and population locations revealed clusters of lower accessibility indices in areas in north east Germany. There was a correlation of urbanity and accessibility up to r = 0.31 (*p* < 0.001). Furthermore, 10% of the population lived in areas with significant clusters of low spatial accessibility for internal medicine and surgery (neurology: 20%). The analysis revealed the highest accessibility for heart failure (AI = 7.33) and the lowest accessibility for stroke (AI = 0.69). The method applied proofed to reveal important aspects of spatial accessibility i.e. geographic variations that need to be addressed. However, for the majority of the German population, accessibility of general inpatient care was either high or at least not significantly low, which suggests rather adequate allocation of hospital resources for most parts of Germany.

## Introduction

The United Nations' 2030 Agenda ‘leaving no one behind’ emphasizes the significance of health equity for health care systems worldwide^1^. However, there are still significant differences in terms of access to healthcare all over the world^2,3^. This also applies to health care in developed countries like Germany^4^. Health inequity has been shown to influence a variety of health-related outcomes such as maternal mortality^5^. Therefore, improving access is a major task for health care systems.

The prerequisite for the improvement of access is a common definition of ‘access’ which represents a multidimensional construct of social, financial, geographical and personal factors. Access in this context describes the process of satisfying medical need by health care resources^6^. In general, there is medical need for health care if there is an objective disease which can be treated effectively^7^. Therefore, the morbidity of a population operationalized by the regional disease burden can be used as a proxy for medical need.

Access has further to be differentiated by potential access and utilized access^8^. There is potential access if the medical need of the population could theoretically be covered by a capable health system, both in terms of time and location^9^. According to Penchansky and Thomas, there are five dimensions of access: availability, accessibility, accommodation, affordability and acceptability^10^. Accommodation, affordability and acceptability describe cultural, personal and financial factors within a health care system. For example, the insurance status (i.e. accommodation and affordability) in Germany has been shown to influence the time to an appointment in the outpatient sector, which in 2019 led to the establishment of appointment service centers by the National Association of Statutory Health Insurance Physicians in Germany^11^. This relationship has also been reported in international studies^12,13^. In addition, the preferences of patients regarding the choice of health care services (‘acceptance’) also play a major role: For the inpatient sector in Germany, it was shown that the personal experience, recommendations by acquaintances or doctors, and the reputation of the hospital are important reasons for the choice of a particular hospital^14^. Finally, among social factors the socioeconomic status influences accessibility and should therefore be considered within the concept of access^15^. It has to be noted that the dimensions described have to be considered co-dependent. In accordance, the geographical dimension ‘availability’ as the number of health care providers and ‘accessibility’ as the travel costs in terms of distance are commonly combined and referred to as ‘spatial accessibility’^9^.

Measurement of spatial accessibility can be accomplished by calculating (1) simple provider-to-population ratios, (2) distances to closest provider or mean distance to a set of providers, (3) population counts within certain catchment areas and (4) gravity models^9^. Predominantly the first three measurements have been used. Derived provider-to-population ratios are used for both inpatient and outpatient sectors in Germany for planning purposes (e.g. Hill-Burton formula which takes into account population size, number of cases, length of stay and use of beds within an administrative area)^4,16^. As for population counts within certain catchment areas, in the 2012 Hospital Report of Germany, it was shown that 73.2% of the population could reach the nearest hospital within 10 min and 97.5% within 20 min^17^. Further developed methods based on gravity models have rarely been used in inpatient care. The necessity of such sophisticated methods is due to the limitations of other more simplified methods, which can only partially reflect the reality of health care delivery^9^. Therefore, there is a research gap concerning the accurate assessment of spatial accessibility of the inpatient sector in Germany both regarding the medical need and the capacity. This study aims to close this gap by investigating the inpatient sector in Germany using sophisticated measures of spatial accessibility accounting for the influence of morbidity, urbanity and socioeconomic status.

## Methods

The study was performed on national level in Germany. Within this study, we pursued two approaches: a population-based approach for the analysis of general spatial accessibility and a morbidity-based approach for a more detailed analysis of spatial accessibility. Both approaches mainly differ in their conceptualization of medical need: Within the population-based approach medical need is defined as the population count per km^2^. Within the morbidity-based approach medical need is defined as the ICD-10 count per km^2^.

### Population-based approach

For each specialty, we retrieved the number of acute care hospital beds as a proxy for capacity and the hospitals’ location (addresses) as of 2017. Long-term care and/or psychiatric care facilities have not been included. Both data (capacity and location) as well as the population count on municipality level were retrieved from the German Federal Statistical Office as of 2017.

For the analysis, we used a km^2^-Grid clipped to the boundaries of the German federal territory. The geographical boundaries of the administrative areas used in this study were retrieved as of 2017 from the German Federal Agency for Cartography and Geodesy. Grid cells whose centroids were more than 500 m away from a car-accessible road were eliminated in order to exclude non-accessible areas such as forests, mountain ranges or water bodies. To each centroid we assigned the relative population size of the municipality according to the total number of centroids within the municipality. Using all centroids and locations of hospitals we calculated an origin–destination matrix with travel time in minutes by car with a maximum catchment size of 120 min. Choosing catchment sizes is arbitrary unless real world data are incorporated. However, due to the lack of data availability, catchment sizes had to be approximated. This issue however is well-known^18^. We chose 120 min as the maximum catchment size, which is in line with the current literature^19–21^.

We used the ‘integrated floating catchment area’ (iFCA) methodology to measure spatial accessibility^18^. This method, which has already been applied in different studies, was chosen since it overcomes several issues of earlier measures of spatial accessibility (e.g. variable distance decay or border crossing)^9,18,22^. Within the iFCA methodology, a variable distance decay function is introduced which more accurately accounts for the distance depending variable utilization of health care (e.g. urban vs. rural catchment areas). In simplified terms, a ratio of hospital capacity and medical need is calculated termed accessibility index (*AI*). The formula for calculating the accessibility index at population location *x* can be represented as follows:

1 $${AI}_{x}=\sum_{y\in ({d}_{xy}\le {C}_{x}) }\frac{{S}_{y}\cdot {f}_{adj}\left({d}_{xy}\right)\cdot {f}_{con}\left({d}_{xy}\right)}{\sum_{x\in ({d}_{xy}\le {C}_{x})}{P}_{x}{\cdot {f}_{adj}(d}_{xy})\cdot {Huff}_{x}}$$

Two steps are necessary for the calculation: In a first step, based at hospital location *y* the hospitals’ capacity (*S*_*y*_) is divided by the summed medical need emanating from all population locations *x* (centroids of the km^2^-grid) with their population size *P*_*x*_ that are within the hospitals catchment area (*C*_*x*_). For this purpose, we used a network analysis to calculate all distances *d*_*xy*_ (in minutes) between population locations and hospital locations. For the analysis we used a street network based on TomTom Multinet data (TomTom N.V., Amsterdam, Netherlands) as of 2016. The applied speed on different roads is determined by the type of the road (for example freeway vs. highway vs. inner city road) and the respective speed limits. Furthermore distance decay was considered to model the decreasing probability of choosing a certain hospital with increasing distances^9,23^. Distance decay is represented by the sigmoid functions *f*_*adj*_*(d*_*xy*_*)* and *f*_*con*_*(d*_*xy*_*),* where the first is adjusted based on the distribution of the distances (median and standard deviation) of the centroids to the first five hospital locations *y*. The adjusted function takes into account the different travel behavior of the population: in rural areas where hospital availability is lower, patients are more willing to accept longer distances compared to urban areas. The second function is identical for all population locations and depends on the catchment area. Again, the choice of the distance function remains artificial but this function was also used successfully in previous studies^18^. *Huff*_*x*_ describes the probability that the medical need is placed at a particular hospital on the basis of the Huff model, which considers competition^24^. The formula of both distance decay functions as well as Huff_x_ is shown in supplementary file 1.

The first step described above results in disaggregated ratios of hospital beds and medical need for each hospital. In a second step, the analysis starts from each population location *x* where all numerical ratios calculated at the first step are summed up accounting for distance decay, as long as the respective hospital location *y* is located within the catchment area. This returns the accessibility index (*AI*_*x*_) at location *x*.

The status-quo of hospital accessibility in Germany can be described with this population-based approach. We further analyzed the spatial accessibility regarding its correlation with the degree of urbanization and the area measurement of socioeconomic status. Data on the degree of urbanization were obtained from the municipal directory information system of the German Federal Statistical Office with the following categories (1) densely populated, (2) intermediate density, and (3) thinly populated. We used the German Index of Multiple Deprivation (GIMD) on municipality level as of 2010, an established area deprivation index based on a British method, as a proxy for the population’s socioeconomic status^25^. We also calculated simpler measures of spatial accessibility, as described in the introduction section: (1) distance to closest hospital and (2) distance to closest five hospitals in order to compare these simpler measures with the gravity model.

### Morbidity-based approach

In order to analyze spatial accessibility of inpatient care more deeply, we selected the two most frequent diagnoses for each specialty (surgery, internal medicine and neurology) based on the three-digit ICD10-Code as of 2017: heart failure (I50: n = 464,724), atrial flutter/fibrillation (I48: n = 313,462), femoral fracture (S72: n = 188,490), gonarthrosis (M17: n = 186,773), stroke (I63: n = 259,594) and epilepsy (G40: n = 147,685). The ICD10-counts were retrieved at district level from the Diagnosis-Related Groups Statistic (DRG) provided by the German Federal Statistical Office. These numbers were then adjusted for age and gender according to the national ICD10-counts and disaggregated on municipality level. From here, the methodology was similar as described above in the population-based approach with the following exceptions: the age-adjusted ICD10-counts of the above mentioned diagnoses were used for *P*_*x*_ and the number of hospital beds for the respective specialty was used for the *S*_*y*_. Hospitals without such hospital beds and/or < 10 reported main ICD10-counts in 2017 were excluded (I48: 7.0%; I50: 12.8%; S72: 18.3%; M17: 20.9%; I63: 9.1%; G40: 17.8%). Hereby we only included hospitals relevant for the respective medical care. Data of hospital visits were retrieved from ‘Quality Reports’ published by the German Federal Joint Committee as of 2017. With this morbidity-based approach the medical need is modelled more accurately according to the initial definition described in the background section^7^.

### (Geo)statistical analysis

For our analysis we used the geographic information software ArcGIS Pro 2.4. (ESRI Inc., Redlands, USA) and the statistical analysis software SPSS version 23 (IBM, Armonk, USA). We performed a hot spot analysis with false discovery rate correction based on the Getis-Ord Gi* statistic. The hot spot analysis is used to identify significant clusters with high and low spatial accessibility (statistical significance is reported with a 99% confidence interval). For the correlation analysis we used Spearman Rho r and for the statistical analysis of mean values we used the Wilcoxon signed-rank test.

## Results

In total, we analyzed 358 million distances between hospitals and population locations.

### Population-based approach

The descriptive results are shown in Table 1. The accessibility indices revealed substantial significant differences (AI: 0.032 vs 0.021; *p* < 0.001) with higher accessibility indices for internal medicine. Furthermore, accessibility was significantly lower (*p* < 0.001) for neurology both in regard to simpler accessibility measures such as distance to closest provider (19.7 min) and the accessibility index (AI: 0.003). Additionally, accessibility for neurology was significantly lower (*p* < 0.001) compared to internal medicine or surgery.

**Table 1:** Overview of results (population-based approach).

	Internal medicine	Surgery	Neurology
**Hospitals (n)**	1109	1063	427
**Hospital beds (n)**	144,642	96,696	25,424
**Mean measures of accessibility [SD]**			
Distance (in minutes) to closest provider	13.5 [6.26]	13.8 [6.4]	19.7 [9.3]
Distance (in minutes) to 5 closest providers	31.1 [3.3]	31.3 [3.5]	36.4 [8.4]
Catchment Area (in minutes)	61.1 [2.6]	61.2 [2.8]	65.3 [6.6]
Accessibility Index	0.032 [0.035]	0.021 [0.022]	0.003 [0.002]

**Figure 1:**
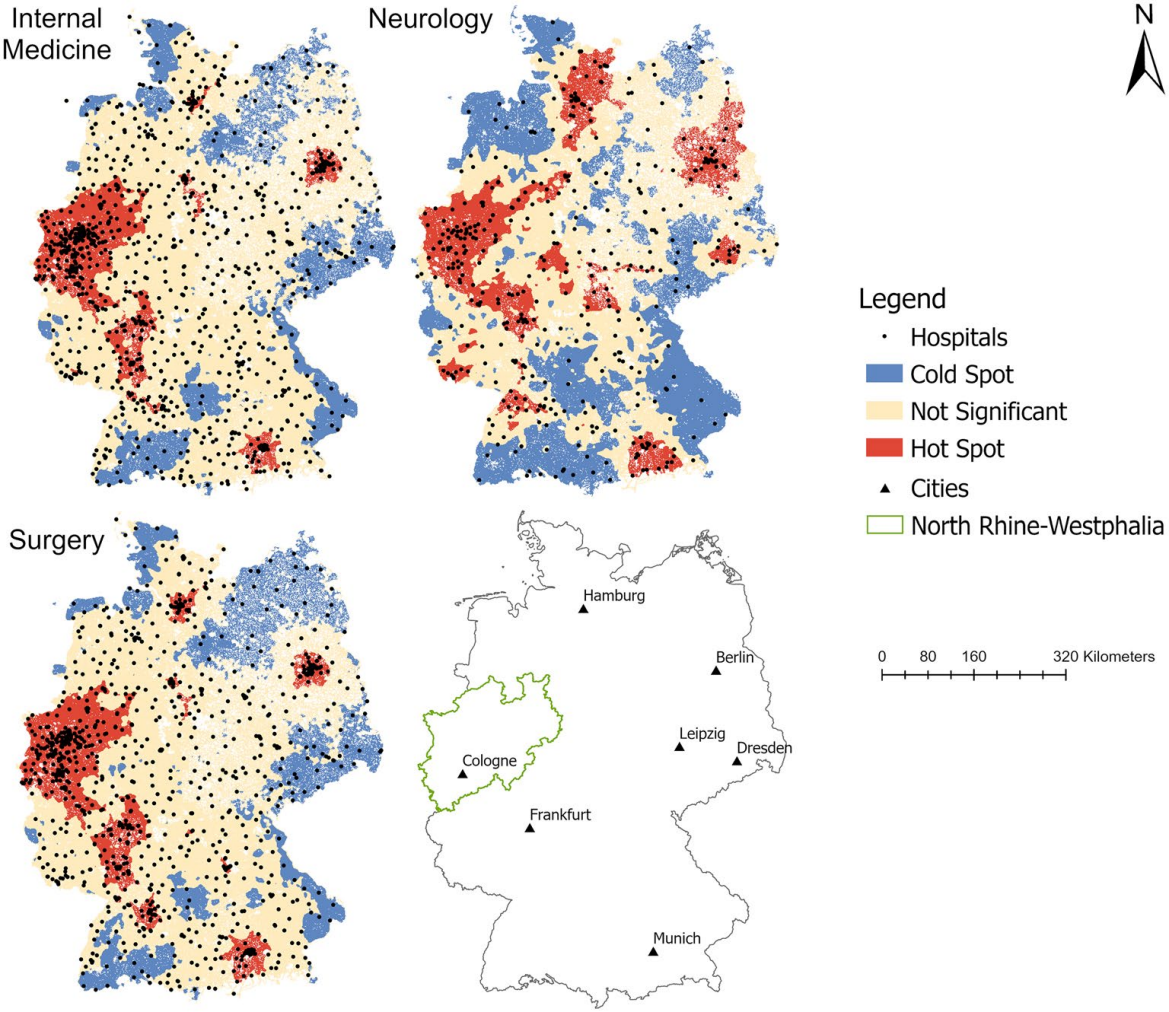
Hot Spot Analysis of accessibility indices (population-based approach). Maps created by the authors with ArcGIS Pro 2.6.1 (https://www.esri.com/de-de/arcgis/products/arcgis-pro/).

Significant clusters of high accessibility indices at urban regions (e.g. Munich and Berlin area) were also present (Fig. 1). However, in other eastern German urban areas like Leipzig or Dresden accessibility was still low. There were significant low accessibility cold spots in the north and the south of East-Germany (except for the Berlin area) compared to West-Germany with a vast area of high accessibility hot spots in the Midwestern area.

In contrast to the significant difference regarding the accessibility indices of internal medicine and surgery as shown above, the relative geographical distribution of the accessibility indices was similar for internal medicine and surgery. This finding is supported by the highly significant positive correlation of both accessibility indices with r = 0.96 (*p* < 0.001). Regarding neurology, this correlation was only r = 0.60 (*p* < 0.001), which translated into a more dispersed spatial distribution of accessibility. However, there were also more cold spots compared to internal medicine or surgery especially in the northwest and the south of Germany. In general, the visual analysis of the spatial pattern for all three specialties suggested a clustering of high accessibility in major urban areas especially in the polycentric urban area in North Rhine-Westphalia and the Berlin and Munich area.

The analysis further revealed that for roughly half of the German population accessibility did not differ regarding internal medicine (51.2% of the population) or surgery (46.4% of the population). Furthermore, only 10% of the population lived in areas with significant clustering of low spatial accessibility for internal medicine or surgery (see Table 2). On the other hand, almost 20% of the population lived in areas with significant clustering of low accessibility in regard to neurology. For all three specialties examined, the proportion of people that lived in an area with significant clustering of high accessibility was around 40%.

**Table 2:** Spatial and demographic results of hot spot analysis.

	Internal medicine	Surgery	Neurology
**Hot spot**			
Area (%)	12.9	14.0	19.3
Population (%)	38.8	43.1	43.4
**Cold spot**			
Area (%)	20.2	22.0	32.4
Population (%)	10.0	10.5	19.5
**Not significant**			
Area (%)	66.9	64.0	48.3
Population (%)	51.2	46.4	37.1

Data in percent in relation to total population and total area.

### Morbidity-based approach

**Figure 2:**
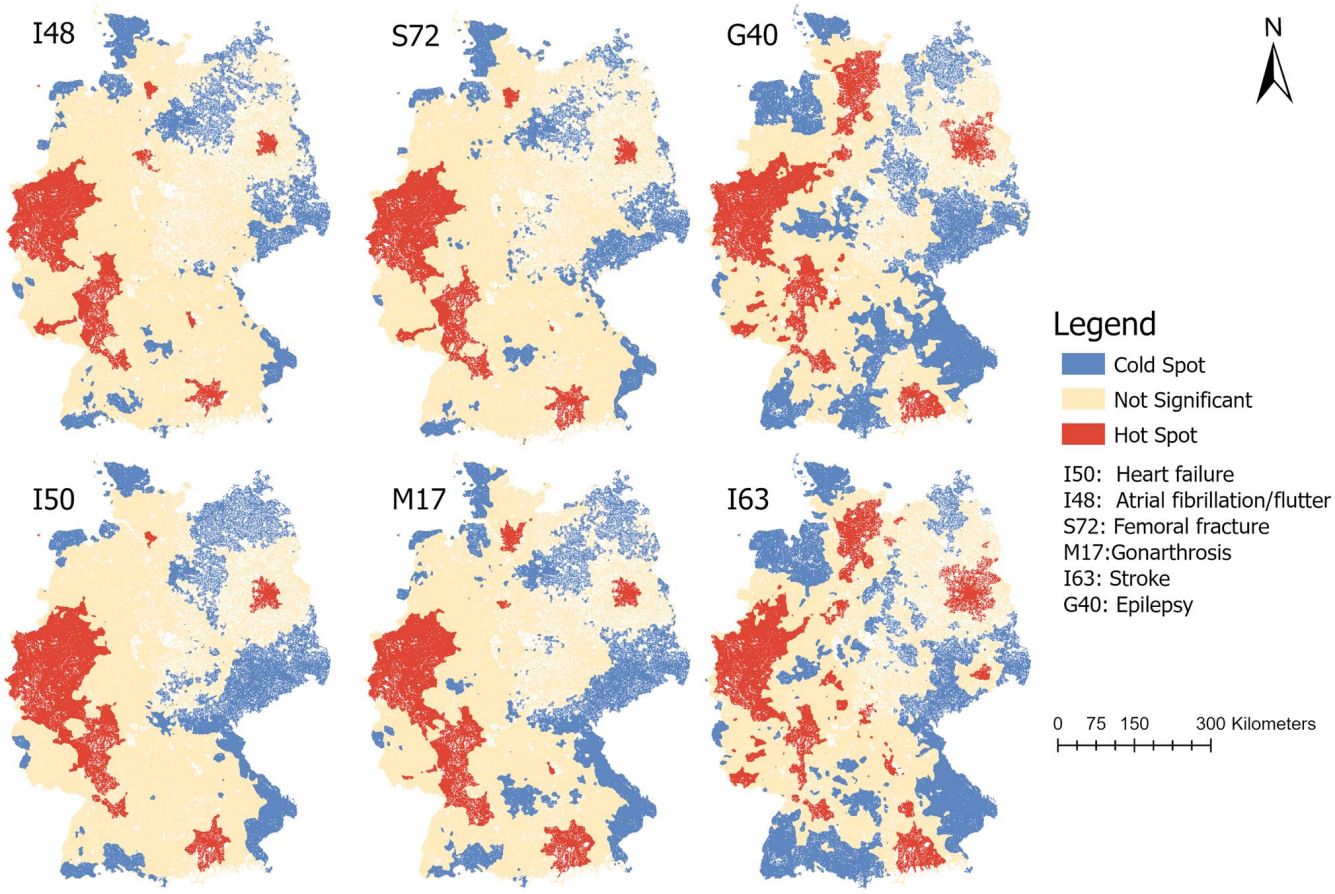
Hot spot analysis of accessibility indices (morbidity-based approach). I50: heart failure; I48: atrial flutter/fibrillation; S72: femoral fracture; M17: gonarthrosis; I63: stroke; G40: epilepsy. Maps created by the authors with ArcGIS Pro 2.6.1 (https://www.esri.com/de-de/arcgis/products/arcgis-pro/).

As shown in Table 3 the more sophisticated spatial accessibility measures revealed the highest accessibility for I50 (heart failure) with AI = 7.33 and the lowest accessibility for I63 (stroke) with AI = 0.69. The spatial distribution of significant hot and cold spots (Fig. 2) of the accessibility indices for all six ICD10 diagnoses revealed a dispersed pattern for stroke (I63) and epilepsy (G40) compared to the other diagnoses.

**Table 3:** Overview of results (morbidity-based approach).

	Internal medicine		Surgery		Neurology	
	**I50**	**I48**	**S72**	**M17**	**I63**	**G40**
**Hospitals (n)**	1031	967	942	911	388	351
**Hospital beds (n)**	141,176	135,740	78,407	76,897	23,294	20,775
**ICD10 Diagnoses at hospital level (n)**	439,671	299,851	168,377	177,203	198,747	108,482
**ICD10 Diagnoses at population level (n)**	463,610	311,035	187,079	182,693	257,528	145,458
**Mean measures of accessibility [SD]**						
Distance (in minutes) to closest provider	13.7 [6.3]	13.9 [6.4]	14.1 [6.5]	14.8 [7.2]	20.0 [9.4]	20.6 [9.6]
Distance (in minutes) to 5 closest providers	31.2 [3.4]	31.4 [3.6]	31.6 [4.1]	32.0 [4.8]	36.9 [8.7]	38.4 [9.8]
Accessibility Index	7.33 [5.99]	7.12 [7.54]	6.71 [7.32]	6.48 [6.85]	0.69 [0.42]	1.02 [0.72]

I50: heart failure; I48: atrial flutter/fibrillation; S72: femoral fracture; M17: gonarthrosis; I63: stroke; G40: epilepsy.

This translated into 20.4% of G40 diagnoses being located in areas with significant clustering of low accessibility and 19.4% for I63 diagnoses. In contrast, regarding both diagnoses, only around 40% of cases were in an area of no significant clustering of high or low accessibility. Regarding atrial flutter/fibrillation (I48), only 8.4% of the cases were in an area of significant clustering of low accessibility. This further translated into a vast majority of I48 diagnoses (91.6%) located in areas where accessibility was either not significantly clustered with low accessibility values or even clustered with high values.

### Comparison of population- and morbidity-based approach

**Figure 3:**
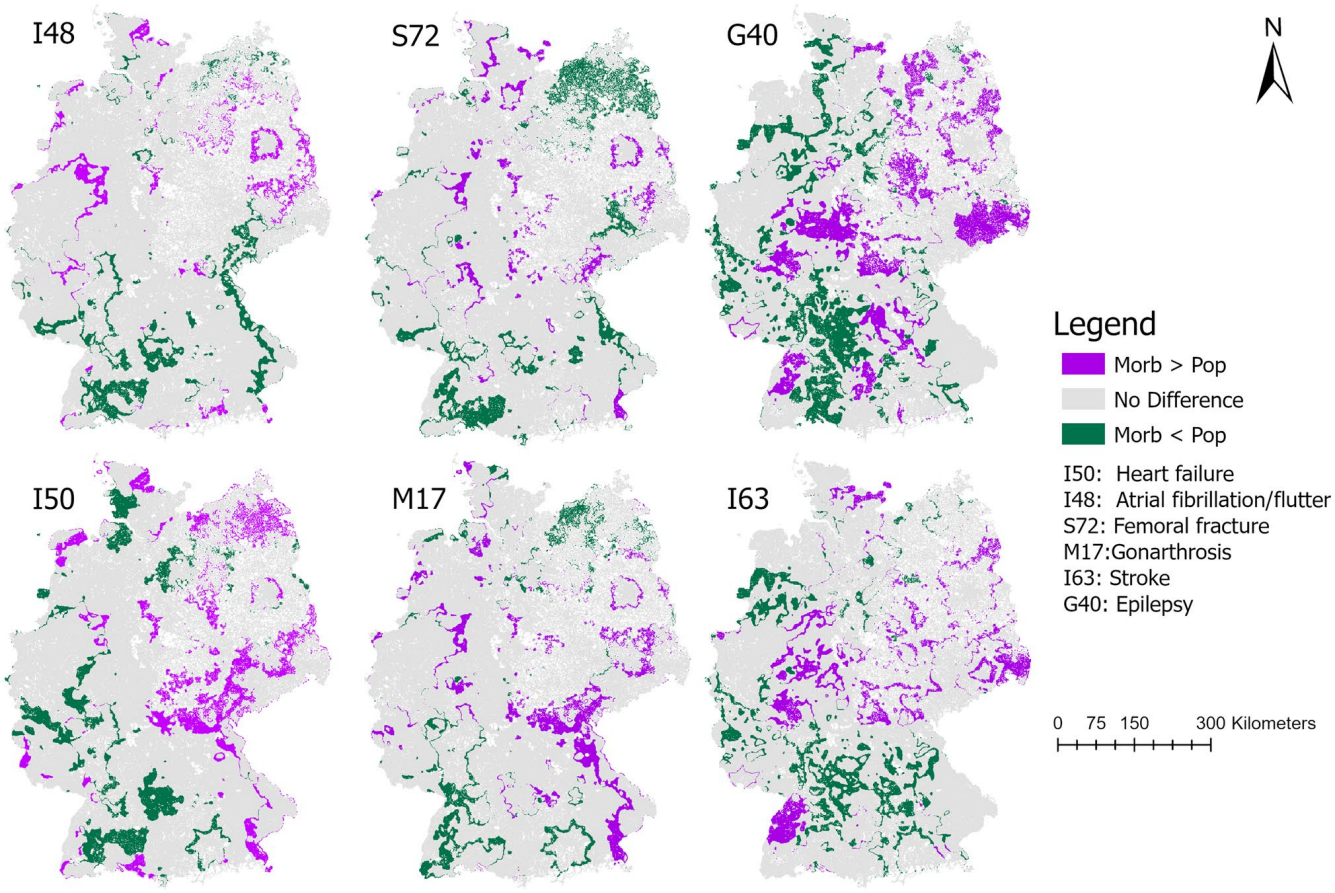
Comparison of population—(pop) and morbidity (morb)—based approach via overlaying both hot spot analyses. Areas not being declared identical (i.e. as hot, cold or not significant spot) are shown. morb > pop: the morbidity-based approach resulted in either 1) a hot spot and the population-based approach in a not significant or cold spot or 2) a not significant spot and the population-based approach in a cold spot; morb < pop: the morbidity-based approach resulted in either 1) a cold spot and the population-based approach in a not significant or hot spot or 2) a not significant spot and the population-based approach in a hot spot. Maps created by the authors with ArcGIS Pro 2.6.1 (https://www.esri.com/de-de/arcgis/products/arcgis-pro/).

The hot and cold spots identified by both approaches differed for the two most frequent diagnoses (morbidity-based approach) and their specialty (population-based approach). In Fig. 3, areas with differing results of the hot spot analysis for both approaches are shown. For the specialty internal medicine (population-based) and heart failure respectively atrial fibrillation (morbidity-based), most of the study area was similarly identified as being a hot, cold, or not significant spot. However, especially around major urban areas (e.g. Berlin or North-Rhine Westphalia) the morbidity-based approach for atrial fibrillation resulted in more areas being declared as hot spots compared to the population-based approach. For heart failure this applied for areas in the East of Germany. For the specialty neurology (population-based) and stroke respectively epilepsy (morbidity-based), the differences were more prominent with more areas being declared differently as hot or cold spots in the morbidity-based approach compared to the population-based approach.

### Correlation of urbanity and area deprivation

The analysis showed a significant but weak relationship of urbanity with accessibility for all analyzed specialties: the more urban the area the higher was the accessibility with a correlation up to r = 0.31 (*p* < 0.001) as shown in Table 4. Regarding the measurement of area deprivation, there was a significantly stronger correlation especially for internal medicine and surgery: the higher the degree of area deprivation the lower was the accessibility with a correlation up to r = − 0.52 (*p* < 0.001). This correlation was less strong for neurology and its associated medical disorders (up to r = − 0.32), but still significant (*p* < 0.001).

**Table 4:** Correlation analysis of accessibility index, urbanity and area deprivation, *p < 0.001; I50: heart failure; I48: atrial flutter/fibrillation; S72: femoral fracture; M17: gonarthrosis; I63: stroke; G40: epilepsy; Urbanity: according to the German Federal Statistical Office**;** Area deprivation: German Index of Multiple Deprivation (GIMD) on municipality level.

	Urbanity	Area deprivation
**Internal medicine**		
General	− 0.29*	− 0.40*
I50	− 0.22*	− 0.49*
I48	− 0.32*	− 0.46*
**Surgery**		
General	− 0.30*	− 0.49*
S72	− 0.31*	− 0.51*
M17	− 0.31*	− 0.52*
**Neurology**		
General	− 0.16*	− 0.12*
I63	− 0.20*	− 0.24*
G40	− 0.22*	− 0.32*

## Discussion

The analysis of 358 million distances between hospitals and population locations revealed significant clustering of spatial accessibility for the general inpatient care in Germany. This is the first time that sophisticated measures of spatial accessibility have been applied to this extent on national level. Earlier approaches on regional or city level cannot account for cross-regional healthcare seeking behavior due to their limited geographical extent. In addition, related cross-regional finance and cooperation issues may be omitted^9^. Therefore, the results provide profound insights into the allocation of hospital resources within the reach of the German healthcare sector. This study showed that only 10% of the population lived within areas of significant clustering of low spatial accessibility for the specialties providing the mainstay of inpatient care (internal medicine and surgery). Therefore, accessibility of the mainstay of inpatient care was either high or at least not significant clustered with low accessibility values for the majority of the German population. Furthermore, spatial accessibility patterns did vary more significantly for neurology: one in five citizens lived in an area with significant clustering of low accessibility. Incorporating morbidity data revealed the highest spatial accessibility for heart failure and the lowest accessibility for stroke. Finally, we found a significant positive correlation of spatial accessibility with urbanity and a negative correlation with the area deprivation. Especially against the background of the immense financial impact of the inpatient care sector Germany, this study may help to improve the allocation process of inpatient care services.

As mentioned above, access is a multidimensional construct composed of a variety of social, financial, geographical and personal factors^6^. In our study we concentrated on the spatial factors of access and therefore omitted financial or personal factors, e.g. that older patients are less likely to travel longer distances^13^. Spatial factors play a major role regarding access since they represent a physical barrier and, thus, are the prerequisite for the utilization of access. As such, our results suggest that this prerequisite is fulfilled for the vast majority of the German population in the fields of internal medicine and surgery. It has also to be noted that due to the differing conceptualization of both approaches used in this study the morbidity-based approach represents a more accurate analysis method. Since the differences between both approaches were more prominent for neurology than for surgery or internal medicine, the morbidity-based approach may be especially helpful for determining the need for health care for smaller medical specialties. These findings may also be partly due to the differing disease prevalence within smaller specialties which may be not as closely correlated to population counts compared to more widespread diseases like heart failure. This is supported by the finding that the absolute ICD-10 counts of heart failure were about three times as high compared to epilepsy. Therefore, the morbidity-based approach can be used in particular for specific issues where whole population counts are not reliable measures of medical need.

In regard to the results being relative measures, the presented study shows that only a minor proportion of the population was living in areas with low accessibility. Consequently, the results could suggest that current hospital planning in Germany allocates hospital resources rather adequately. However, defining low and high accessibility is still an unresolved issue. Areas with high spatial accessibility could be caused by a care level that simply exceeds the medical need. On the other hand, the lowest level of accessibility identified could be the appropriate level of care, especially since Germany has a relatively high bed capacity. The definition of an accessibility threshold needs a political and public debate about the level of care that is desired. The presented results can facilitate this debate leading to a common understanding of health care accessibility. It further must be noted that in our study, we did not differentiate between the level of acute care hospitals (e.g. university hospital vs. regional hospital). However, all included acute care hospital can provide the necessary care for the modelled health care need in this study (e.g. atrial fibrillation and/or stroke) and higher levels of acute care hospitals (e.g. university hospitals) are only specifically needed for complicated or rare diseases which have not been modelled in this study.

The shown geographical variation of spatial accessibility is in line with the current literature^3,26,27^. In our study, there was a positive correlation between urbanity and spatial accessibility. Urban–rural gradients are global phenomena and our results are in line with corresponding observations^28^. The impact of both urbanization and socioeconomic status on access to healthcare has just recently been shown at an international level^3^. The significant impact of the socioeconomic status using a measure of area deprivation has also been revealed in our study. In Germany, it has been shown that people with a low socioeconomic status are increasingly disadvantaged in terms of medical access^29^. However, this relationship has mainly been stated using the non-spatial factors of access such as the organization or the affordability of care. Based on our results the spatial factors of access showed a significant relationship with area deprivation. For example, Greiner et al. also exhibited that area deprivation is negatively associated with accessibility of health care providers^30^. Hence, to improve accessibility, reducing area deprivation should be taken into account. Certainly, as a limitation it has to be acknowledged that the GIMD was as of 2010 and therefore may not represent very recent area-based socioeconomic changes. However, we do not expect major changes between 2010 and 2017.

In this study, we applied a morbidity-based approach to measure medical need. As stated in the introduction section, the direct measuring of medical need is virtually impossible due to its latent construct. Therefore, approximates have to be used. We considered the treatable morbidity (i.e. ICD-counts on municipal level) as the optimal way to operationalize medical need which is in line with current literature^4,7,31^. Consequently, the morbidity-based approach has to be considered more valid than the population-based approach due the above-mentioned reasons. However, using ICD codes generated in the inpatient sector as the measure of medical need could be biased by up-coding practices meaning that the ICD code used differs from the actual diagnosis. Furthermore, physician induced demand could bias the medical need that is measured. Both issues need to be noted and could be overcome in future studies by using outpatient diagnosis.

Due to the authorization of some hospitals to provide ambulatory care, the ambulatory sector has to be considered within the concept of access to health care despite major differences regarding planning and organization. Concentrating solely on one sector omits interdependence and its influence on health care access.

The methodology used (iFCA) is based on the established two-step floating catchment area method (2SFCA)^32^. However, there are multiple derived methods that have been used so far (e.g. enhanced (E)2SFCA method, kernel density function (KD)2SFCA method or enhanced variable (EV)2SFCA method)^18^. Uncertainty remains regarding the methodology because there is no consensus on which method offers the best results. This also applies to the choice of the maximum catchment area (120 min). Even though this catchment area has been used in a similar study in the inpatient sector, it remains arbitrarily and was mainly applied to limit computation time^27^. Due to the variable distance decay function used in the iFCA-method, the average catchment area varied between population locations (e.g. urban vs. rural) resulting in average catchment areas around 60–65 min. For the outpatient sector, catchment areas are often reported to be much smaller due the higher availability of health care providers^21^.

Compared to the outpatient sector, the general approach of the iFCA method for the inpatient sector is similar. However, there are two main differences regarding the approach of measuring spatial accessibility for inpatient versus outpatient sector: the catchment area and the definition of need for health care. The catchment area of inpatient care is usually larger compared to outpatient care, even though outpatient care encompasses a variety of health care services (e.g. primary care vs. specialized care) and catchment areas vary across these services^21^. The need for inpatient care usually implies more serious and/or more complex diseases compared to the outpatient sector. In our study the need for health care was based on the ICD-Codes that have been used in real hospital cases. ICD-Codes, however, can also be applied in the outpatient sector since the ICD-codes do not differentiate between the health care sector. If ICD-Codes are to be applied to model the need for outpatient care, only those that represent real outpatient cases should be utilized^4^.

The iFCA method used in this study is based on established methods and has been shown to return valid results^18,22^. Using an estimation of grid-based population densities as applied in this study, accounts for differences in urban and rural population densities. To a lesser degree it also accounts for different land uses. Land uses like residential areas, industrial areas, agricultural areas, or recreational areas differ in the degree of traffic connection: Residential areas are areas which have a very good traffic connection (i.e. car accessible roads). Recreational areas (like forests or water bodies) or agricultural areas are unlikely to have the same degree of traffic connection compared to residential areas. Industrial areas, however, often have a good traffic connection. Therefore, our approach may overestimate the population density in industrial areas. Still, land use has been partly accounted for in our approach. Another factor to be considered is the variation of population distribution over time. This spatio-temporal accessibility issue, which also includes commuting effects, has been addressed by several studies showing that the population sizes can vary greatly over time^33^. Furthermore, the mode of transport (e.g. car or public transport) has an influence on accessibility, especially if the choice is limited because of physical constraints^34^. In this study, we only used driving by car as mode of transport and therefore omitted its multimodal reality. Multimodal models of spatial accessibility have already been used within health services research^34^. However, such analyses require the availability of certain transport data, which are not readily available in Germany so far. Omitting multimodal transport (e.g. public transport) may especially limit the conclusion in urban areas, where public transport is a major mode of transport compared to more rural areas. Finally, despite using common statistical spatial analysis tools we acknowledge that there are different approaches to spatial statistics. Although density analyses would also be possible (for example, using the kernel density function), they do not return information about significant clusters and, thus, were not suitable for this study. In summary, the method applied in our study has been applied in different settings before, however, due to the novel application of this method within the inpatient care sector in Germany on national level, the insight provided by this study can help to better understand the differences regarding spatial accessibility of inpatient care in Germany.

## Supplementary information

Supplementary Information.

## Data Availability

The data that support the findings of this study are available from the German Federal Statistical Office but restrictions apply to the availability of these data, which were used under license for the current study, and so are not publicly available. Data are however available from the authors upon reasonable request and with permission of German Federal Statistical Office.
