# Supplementary material for: Spatial accessibility of general inpatient care in Germany: an analysis of surgery, internal medicine and neurology
Source: Sci Rep. 2020 Nov 5;10:19157. doi: 10.1038/s41598-020-76212-0 (PMC7645718; doi:10.1038/s41598-020-76212-0)
Supplement: Supplementary file 1 — Supplementary Information. [file 41598_2020_76212_MOESM1_ESM.docx]

**Spatial accessibility of general inpatient care in Germany – An analysis of surgery, internal medicine and neurology**

Jan Bauer*^1^; Doris Klingelhöfer^1^; Werner Maier^2.^; Lars Schwettmann^2.3^; David A. Groneberg^1^

^1^Division of Health Services Research, Institute of Occupational Medicine, Social Medicine and Environmental Medicine, Goethe University Frankfurt, Theodor-Stern-Kai 7, 60590 Frankfurt

^2^Institute of Health Economics and Health Care Management, Helmholtz Zentrum München, German Research Center for Environmental Health (GmbH), Ingolstädter Landstr. 1. 85764 Neuherberg, Germany

^3^Department of Economics, Martin Luther University Halle-Wittenberg, 06099 Halle an der Saale, Germany

**Supplementary File 1**

Distance decay was considered to model the decreasing probability of choosing a certain hospital with increasing distances.^1,2^ Distance decay is represented by the sigmoid functions *f_adj_(d_xy_)* and *f_con_(d_xy_).* Both functions are based on the logistic cumulative distribution function of the logistic function. *f_adj_(d_xy_)* is adjusted based on the distribution of the distances (median and standard deviation (SD)) of the centroids to the first five hospital locations *y*.

Equation S1:

$$f_{adj}\left( d_{xy} \right)=\frac{1+e^{-\frac{\left( Median \right)*\pi}{SD*\sqrt{3}}}}{1+e^{\frac{\left( d-Median \right)*\pi}{SD*\sqrt{3}}}}$$

The adjusted function takes into account the different travel behavior of the population: in rural areas where hospital availability is lower, patients are more willing to accept longer distances compared to urban areas.

The second function is identical for all population locations and depends on the catchment area. We choose a catchment area of C=120 minutes. Based on this catchment area, a standard deviation (SD_con_) has to be used that results in *f_con_(C)*=0.01 (i.e. a weight value of 0.01). This cut off value has been used as a critical value approaching zero ^3,4^. This decay function is used to fit all decay functions within the chosen maximum catchment area and guarantees its adherence.

Equation S2:

$$f_{con}\left( d_{xy} \right)=\frac{1+e^{-\frac{\left( C/2 \right)*\pi}{SD_{abs}*\sqrt{3}}}}{1+e^{\frac{\left( d-C/2 \right)*\pi}{{SD}_{abs}*\sqrt{3}}}}$$

*Huff_x_* describes the probability that the medical need is placed at a particular hospital y compared to all other hospitals *z* on the basis of the Huff model, which considers competition.^5^

Equation S3:

${Huff}_{x}=\frac{S_{y}\cdot f_{adj}\left( d_{xy} \right)}{\sum_{z\in(d_{xz}\leq C_{x})} S_{z}{\cdot f_{adj}(d}_{xz})}$

The speed limits used to calculate travel distances are based on the regulatory speed limits for the respective roads. The TomTom Multinet data used, distinguished nine functional road classes from motorway to local roads of minor importance.^6^ For each functional road class speed limits were applied ranging from 120 km/h (motorway) to 15 km/h (local road of minor importance) also depending on whether the road runs through a built-up area. Using such functional roads is also suggested by the European Commission.^7^

References

1. Weinhold, I. & Gurtner, S. Understanding shortages of sufficient health care in rural areas. *Health Policy (New. York).* **118**, 201–214 (2014).

2. Guagliardo, M. F. Spatial accessibility of primary care: concepts, methods and challenges. *Int J Heal. Geogr* **3**, 3 (2004).

3. Kwan, M. Space-time and integral measures of individual accessibility: a comparative analysis using a Point-based framework. *Geogr Anal* **30**, 191–216 (1998).

4. Wang, L. Immigration, ethnicity, and accessibility to culturally diverse family physicians. *Heal. Place* **13**, 656–671 (2007).

5. Huff, D. Defining and Estimating a Trading Area. *J Mark.* **28**, 34–38 (1964).

6. TomTom International BV. What are functional road classes? https://developer.tomtom.com/traffic-stats/support/faq/what-are-functional-road-classes-frc (2020).

7. European Comission. Road Classification. https://ec.europa.eu/transport/road_safety/specialist/knowledge/road/designing_for_road_function/road_classification_en (2020).
